# Exploring life’s hidden majority: microbial dark matter symposium highlights

**DOI:** 10.1128/msphere.00587-25

**Published:** 2026-07-10

**Authors:** Zachary N. Flamholz, Sayali A. Mulay, Victor Leshyk, J. Gregory Caporaso, Jonathan A. Eisen, Libusha Kelly, Karen G. Lloyd, Magdalena R. Osburn, Mircea Podar, Simon Roux, S. Aaron B. Regberg, S. Emil Ruff, Braden Tierney, Scott Tighe, Elizabeth Trembath-Reichert, Kasthuri Venkateswaran, Tanja Woyke, Kristopher M. Locken, Haley M. Sapers, Katrine Whiteson

**Affiliations:** 1 Albert Einstein College of Medicine, Bronx, New York, USA; 2 University of California 8788, Irvine, California, USA; 3 Northern Arizona University 3356, Flagstaff, Arizona, USA; 4 University of California 8789, Davis, California, USA; 5 University of Southern California 5116, Los Angeles, California, USA; 6 Northwestern University 3270, Evanston, Illinois, USA; 7 Oak Ridge National Laboratory 6146, Oak Ridge, Tennessee, USA; 8 U.S. Department of Energy Joint Genome Institute, Lawrence Berkeley National Laboratory 1666, Berkeley, California, USA; 9 NASA Johnson Space Center 43834, Houston, Texas, USA; 10 The Marine Biological Laboratory, Woods Hole, Massachusetts, USA; 11 The Two Frontiers Project, Fort Collins, Colorado, USA; 12 University of Vermont 2092, Burlington, Vermont, USA; 13 Arizona State University 7864, Tempe, Arizona, USA; 14 NASA Jet Propulsion Laboratory, Pasadena, California, USA; 15 Zymo Research Corporation 58267, Irvine, California, USA; Shenzhen Institute of Synthetic Biology, Chinese Academy of Sciences, Shenzhen, China

**Keywords:** metagenomics, microbial dark matter, meeting highlight

## Abstract

The Microbial Dark Matter Symposium held on August 28–29, 2025, in Laguna Beach, Orange County, CA, convened a multidisciplinary group of scientists to address the vast unknowns in microbial life—from uncultured taxa and uncharacterized proteins to elusive viruses and spacefaring microbes. Set against a scenic coastal backdrop, the symposium highlighted advances in single-cell genomics, proximity ligation sequencing, and artificial intelligence-ready bioinformatics, while also probing the limits of microbial persistence, metabolism, and ecological distribution. Sessions explored microbial dark matter from multiple dimensions: cultivability, where new strategies are enabling recovery of elusive microbes; functional ambiguity, where metagenomic dark zones are illuminated by computational annotation; and genomic representation, where single-cell methods bridge gaps left by shotgun community sequencing. Researchers shared breakthroughs in identifying atmospheric microbiomes, “dark oxygen” production in groundwater ecosystems, and microbial survival on the International Space Station. The symposium emphasized integration of methods, disciplines, and ecosystems, advancing a collective push to illuminate the microbial dark matter on Earth and beyond. By highlighting emerging tools, pressing questions, and cross-domain insights, the symposium underscored the need for collaborative, open, and adaptive approaches to study the microbial unknown. The meeting marks a pivotal moment in microbiology, where cultivating knowledge of the uncultivated promises transformative understanding of life, everywhere.

## INTRODUCTION

## DARK MATTER IS THE INVISIBLE MATTER THAT MAKES UP 80% OF THE MATTER IN THE UNIVERSE

“Physicists did not discover dark matter by going out to look for dark matter”, began particle physicist Dr. Daniel Whiteson, as he opened the 2025 Microbial Dark Matter Symposium, “physicists want to know what the world is made of.” Similarly, in microbial ecology, we are interested in the life the world is made of. In attempting to answer this question, one encounters microbial dark matter (MDM). Perhaps the most striking temporal juxtaposition of fundamental discovery and foundational exploration in the field of microbial ecology occurred over 8 months in the mid-1970s. In July 1976, Viking 1 landed on the surface of Mars (1), the first, and only, life detection mission sent beyond Earth. Yet it was not until 8 months later that we began to comprehend the astonishing diversity of life on Earth. In March 1977, the first hydrothermal vents were discovered off the coast of Ecuador (2). There were no biologists on that initial expedition. At the time, it was inconceivable that entire ecosystems, fueled by subsurface energy sources, could be present at the bottom of our oceans; indeed, as Viking’s astrobiological remit showed, it was considered more probable that life might be present in the frozen deserts of Mars. Since that pivotal discovery and profound shift in our collective understanding of evolution and diversity, we now estimate that 70%–90% of all bacterial and archaeal cells on Earth persist in the subsurface (3, 4), sustained without light through chemolithotrophic metabolic diversity. Whether in exotic unexpected extreme environments or well-studied human samples, much of the estimated microbial biodiversity and metabolic function remains unexplored as microbial dark matter.

**Fig 1:**
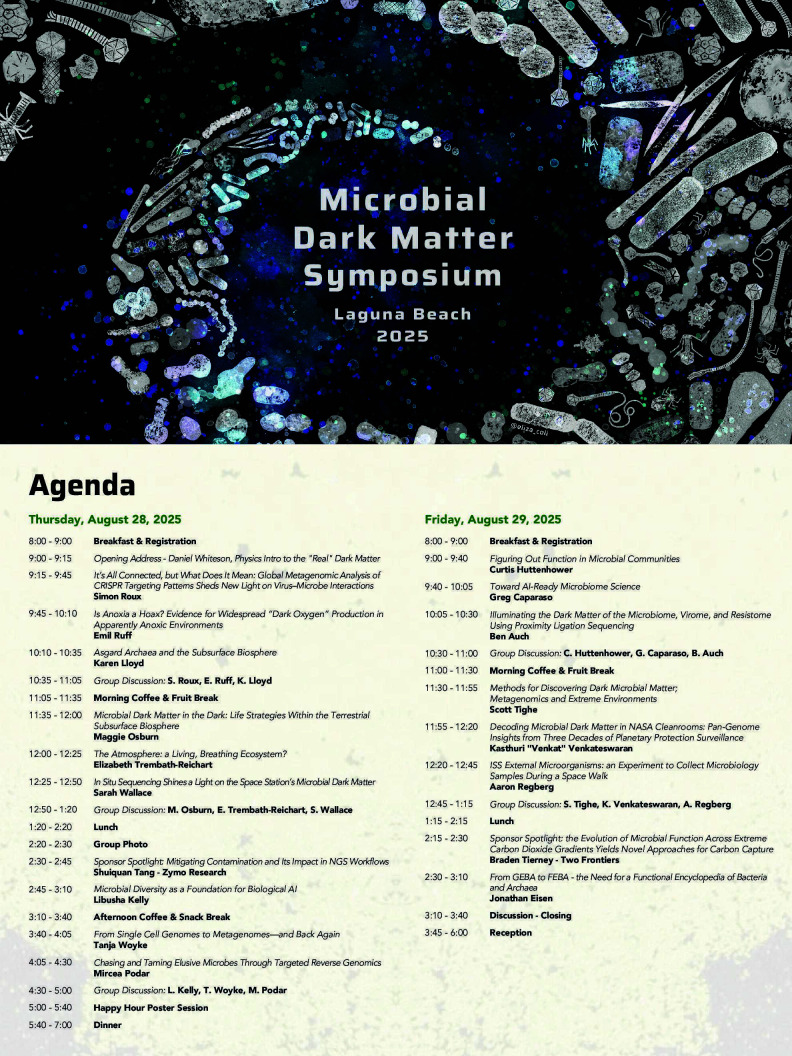
Agenda for the 2025 Microbial Dark Matter Symposium.

Tucked in the sun-kissed canyons of Laguna Beach, just a breeze away from the Pacific Ocean, The Ranch hosted what one speaker called a “Festival of Microbiology.” The Microbial Dark Matter Symposium brought together leading academic and industry researchers, government stakeholders, and early-career scientists fostering cross-domain dialog on the microbial unknown (see agenda in Fig. 1). The symposium, rich in cutting-edge science and captivating storytelling, offered a snapshot of microbial research that is radically redefining our understanding of biological systems, their ecological functions, and their evolutionary histories and trajectories. Over the course of 2 days, attendees shared their approaches, findings, insights, challenges, frustrations, and aspirations for engaging with MDM. The scope of the science was as vast as the biosphere and even beyond. It was clear that while we observe, physically and computationally, more life than ever before, there is much work to be done.

## WHAT DO WE MEAN BY MICROBIAL DARK MATTER?

**Fig 2:**
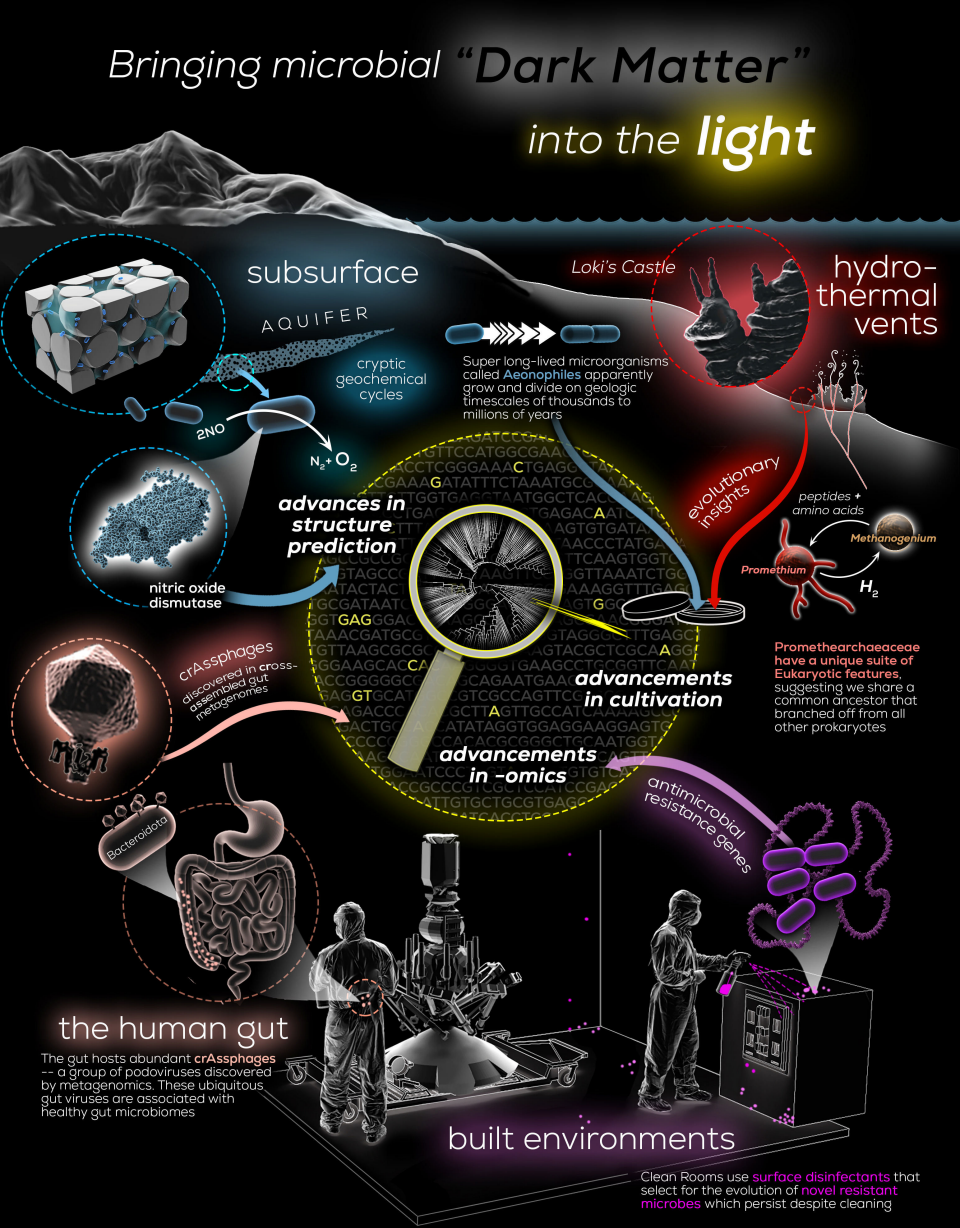
Bringing microbial dark matter into the light. Advances in cultivation, -omics, and computation are revealing previously uncharacterized microbial diversity across multiple environments, including subsurface aquifers with cryptic geochemical cycles, deep-sea hydrothermal vents hosting ultra-slow-growing aeonophiles, the human gut with its abundant crAssphages, and spacecraft clean rooms harboring novel antimicrobial-resistant microbes.

Each speaker was asked in advance of their talk to recall their first encounter with MDM. One after the other, they recounted stories of wonder and confusion at the mystery they saw as young scientists. For many, it was microbial diversity found in their field site, from salt mines to an obsidian pool (5, 6) to non-aqueous phase liquid at an oil-contaminated aquifer (7) and even the “pan pudding” of a home heating, ventilation, and air conditioning unit (8). For others, it was encountering the vast catalog of genomic content with unknown function, even in well-studied model organisms like *Escherichia coli*. Some talked about the inadequacy of 16S rRNA gene-based phylogeny and the difficulty in designing universal primers as known microbial diversity exploded. Dr. Mircea Podar recalled a lecture in the early 2000s by Dr. Karl Stetter on the discovery of Nanoarchaeota (9), which, “the so-called universal primers had no chance in hell of amplifying.” Presenters later in the program, aware of the prompt, scoured their emails and drives for the earliest mention of the term in their own lexicon and shared reflections on upwards of 20 years of thinking about MDM (10). A few questioned its use in describing the phenomenon, explicitly recalling a decade-old defense of the term made by one speaker, Dr. Karen Lloyd (11). The light-hearted recollections reflected the sincerity, passion, and dedication of the community to understanding life in all its forms. Several key examples are depicted in Fig. 2, from understudied deep aquifers with cryptic geochemical cycles, deep-sea hydrothermal vents hosting ultra-slow-growing aeonophiles, the human gut with abundant crAssphages only discovered through metagenomics (12), and spacecraft clean rooms harboring novel antimicrobial-resistant microbes.

Different interpretations of the term MDM, as evidenced by these anecdotes, carried through the prepared presentations. Dr. Kasthuri Venkateswaran of National Aeronautics and Space Administration’s (NASA) Jet Propulsion Laboratory addressed the term directly while discussing his work on planetary protection, arguing that there is no such thing as MDM and any “darkness” is due to our inability to shine the right light. He outlined common definitions of MDM:

1. MDM refers to the vast majority of microorganisms in nature that remain unable to be cultured, lysed, or characterized and remain largely unknown despite their ubiquity and ecological significance.
2. MDM in DNA sequencing refers to genetic material from uncultivated phylogenetically uncharacterized microorganisms, revealed through metagenomics and single-cell genomics, which expands the microbial tree of life but remains functionally unvalidated in culture.
3. MDM refers to phylogenetically unassigned DNA sequences that resist classification, not due to irrelevance or “junk” but because they represent unexplored lineages and highlight the limitations of existing genomic databases.

*Nota bene:* MDM must be distinguished from sequences that remain unassigned due to technical or analytical limitations. Whereas MDM reflects genuine biological novelty and uncultivated lineages, many unclassified sequences arise from short read lengths, sequencing errors, low coverage, and database gaps, underscoring the need for cautious interpretation of microbial “dark” DNA. In fact, early virome sequencing data sets with large numbers of unmappable reads were assumed to be technical error rather than biological in origin, until we better understood the incredible diversity of viruses (13–16).

From Dr. Venkateswaran’s perspective, each characterization represents a limitation of our methods, concluding, “this MDM that we are all defining can be changed over time with better technology tomorrow.” There was general agreement in the room at his statement, with many in the room actively pushing that technological frontier.

At the same time, the scale of the microbiology we do not understand was apparent throughout the meeting, possibly shifting the dark matter analogy away from a lack of understanding of the phenomenon to the sheer amount of it (physicists estimate that only 5% of matter is the physical matter we are aware of, while the rest is dark matter or dark energy). With phylogeny as the anchor to understanding microbial diversity and genomic sequencing as the data engine for sampling that diversity at scale, we have identified millions of Earth’s microbial taxa, and the pace of discovery is not slowing down (17–19). In the first talk of the meeting, Dr. Simon Roux walked through 20 years of viromics. He emphasized the enormity of viral diversity collected from bulk sequencing and the imperative to better characterize these viruses with new approaches (20). Keynote speaker Dr. Jonathan Eisen, an evolutionary biologist and an avid birder, highlighted the need for the community to shift focus from databasing to characterizing. He suggested a field guide model similar to what bird watchers and other naturalist communities use for cataloging (21). By aggregating properties like biogeography, means of identification, functional diversity, niche, and *in situ* function, he suggests one could interpret normal and abnormal encounters with a microbe. He called for the creation of a “Functional Encyclopedia of Bacteria and Archaea (FEBA)” project to be the functional extension of the “Genomic Encyclopedia of Bacteria and Archaea” (GEBA) project (22). Such a resource would have utility for identifying emerging pathogens, microbial forensics, tracking climate change, vaccine design, managing antimicrobial resistance, and likely many other areas.

While not yet an actual resource, it is through the lens of such a guide that one can appreciate the science presented at the 2025 Microbial Dark Matter Symposium. In the terrestrial subsurface, geochemical context produces distinct, stable microbial niches even within a relatively small distance (23, 24). The diversity of the aerosphere microbiome correlates with time of day and the physical properties that shift throughout the day (e.g., reference 25). On spacecraft, human-associated microbes appear where you might expect, and sometimes not expect, like on a spacewalk. Ancient metabolically active archaea, termed aeonophiles, were found by searching frozen subsurface waters (26). Aerobic microbes and oxygen-producing pathways are widespread in apparently anoxic environments (27). Whether or not the microbes identified can be cultured, the vast majority of them never will be due to their sheer number and the unique challenges required to culture each new taxon. Triangulating the role a microbe plays in its environment, through interrogation of its genome, the structure of its community, and its ecological context, is crucial in realizing the vision of a field guide for microbes.

In chasing MDM, every speaker noted the difficulties with such triangulation. Here, we will discuss themes that emerged from the prepared presentations and discussion sessions. Each topic is headlined by a direct quote from the conference, many of which Dr. Eisen himself recorded and shared at the end of his talk to spur conversation.

## MAIN THEMES

### “Anything is better than nothing”

Those looking for microbes in extreme environments shared a common challenge: low biomass. From the Siberian permafrost and the deep subsurface to NASA clean rooms and the air around us, the low density of microbes in these environments limits our ability to collect and sequence them and study their activity. Multiple speakers were measuring sample material in femtograms (10^−15^). Scott Tighe talked about his work on the Extreme Microbiome Project and outlined tactical elements of sampling at low biomass: (i) collection, (ii) preservation, (iii) DNA extraction, (iv) DNA amplification, and (v) sequencing. Each element requires thoughtful preparation and bespoke design for the target collection. Speakers highlighted examples of innovations they have employed at each of these steps. A DNA repair step unlocked partially damaged extracellular DNA as a source of additional genetic material (28). Cocktails of lytic enzymes (e.g., PreCR Metapolyzyme and Exopolyzyme by Millipore Sigma) were helpful in extracting greater DNA yield by degrading different cell wall components and extracellular matrices (29). Amplification with primary template-directed amplification outperformed multiple displacement amplification for resolving single-cell genomes in environmental microbiomes (30). Sometimes the solution was to access more surface area or volume, like filtering 72,000 liters of air per sample. Getting anything in your tubes is the first and sometimes most difficult step in studying MDM.

### “We don’t have monkeys running around in the clean room”

Dr. Shuiquan Tang, director of microbiomics at Zymo Research, opened his talk with a statement the experimentalists in the room knew too well, “If you go after MDM, you need to worry about contamination.” A recent consensus paper outlines best practices for avoiding contamination in microbiome sampling (31) and discussion at the meeting provided real-world experience and examples to match the guidance. Sample controls are critical to detect contamination introduced by the people, equipment, and surroundings where sampling occurred. Reagent controls are used for possible contamination in the wet lab’s sample processing steps. The terms kitome and extractome, referring to cellular or nucleic acid contaminants present in commercial molecular biology kits, were used multiple times reflecting familiarity and consideration of issues with reagent contamination. Preservation buffer is a necessary component of sample collection when the sampling site is far from the lab, but reliably DNA-free buffers remain an aspiration. Finally, there are bioinformatic methods for identifying and removing contamination, but it is crucial to use suitable databases, dedicated tools, and appropriate sequence and abundance thresholds; distinguishing MDM from contamination necessitates a nuanced and hands-on approach.

Microbes and contamination was the focus of another group of talks at the meeting but from a different vantage point. At NASA, the objective of Forward Planetary Protection is to prevent biological forward contamination during interplanetary missions. To achieve this objective, NASA constructs environments that are meant to be clean of microbial life and monitors them for contamination. Microbes found in these clean rooms can be newly identified and extremotolerant due to the harsh conditions of the environment (32). These species were not present in large sequence databases, like IMG/M, spurring a discussion about low abundance and thresholds in microbiome bioinformatic workflows. Another microbial concern at NASA is contamination that endangers crew safety inside spacecraft (33). Dr. Sarah Wallace shared how, using culture-independent sequencing, scientists can monitor crew health and identify high-risk surfaces and areas in real time (34). Linking these projects, Dr. Aaron Regberg discussed an experiment conducted on a recent spacewalk (35) investigating whether microbes are leaking from internal human environments into space. In the search for extraterrestrial signs of life, such leakage could spoil material collected from some distant planet. When one day evidence of past or present life returns from space, consideration of contamination will be crucial to interpreting the finding. The MDM community brings important expertise to this issue.

### “You can just get Everclear”

[Everclear is a U.S. brand of grain alcohol sold at ~95% ethanol (190 proof), available in U.S. liquor stores in some states and sometimes used by field researchers as an accessible substitute for laboratory-grade ethanol for nucleic acid preservation when commercial reagents are unavailable*.*]

Novel diversity requires novel approaches and ingenuity often at the extremes of life in exotic locations. Many talks featured solutions engineered in the field to identify, recover, and protect a sample. For example, ethanol is non-toxic for sample storage and shipping. It can preserve not just nucleic acids but also metabolites, and can often be procured in places where commercial reagents are harder to find. Dr. Braden Tierney talked about his work leading the Two Frontiers Project, a research initiative founded to translate naturally occurring microbial diversity and physiology into solutions for human challenges (36). He highlighted a virtuous cycle where his team can sample, sequence, enrich, and repeat in the field to return from the expedition with the microbes they sought. Working on methods for carbon capture, the process resulted in the discovery of a cyanobacterium that sinks, named Chonkus for its bulky appearance, potentially alleviating a hurdle in bioproduction related to biomass concentration (37). Portable sequencing was noted across many talks to be a significant technological advance for studying MDM. The group curates *The Two Frontiers Handbook*, a guide to field research and best practices.

### “Nothing is unculturable but pure is not always pure”

One refrain of the meeting, especially among veteran scientists, was that with thought, creativity, and fortitude, any microbe can be studied in a lab. In the 1970s, environmental microbiologists often encountered “the great plate anomaly,” where little could be cultured from samples with great diversity of microbes as viewed directly with a microscope. It is now true that many bacteria are culturable with existing methods (38–40), yet some MDM require innovation. It took a decade to culture an Asgard archaeon, which required co-culture with a methanogen. The achievement was worth the wait given its implication for eukaryogenesis (41). Increasingly, novel taxa require co-culture or consortia due to obligate symbiotic relationships. Looking at a scanning electron micrograph of a ectobiont SR1 bacterium, *Rotundibestia catena*, Dr. Mircea Podar stated, “This biology you will not see in sequencing alone” as he then described an amino acid pulse chase experiment used to understand how the minimal bacterium divides. If anything can in fact be cultivated in the lab with enough dedicated effort, the question becomes how to decide what to study (42).

One topic that sparked a lot of discussion was enrichment cultures, which are crucial to the study of MDM. An enrichment culture is a method used in microbiology to favor the growth of particular microbes from a mixed community but is not a pure culture. Meeting participants voiced their desire for more organized and standardized sharing of enrichment cultures, potentially through culture collections. A “dark culture collection” or “dark web of enrichments” was suggested for grassroots sharing with the path toward a recognized standard for enrichment cultures felt to be out of reach by some. If the barrier between enrichment and pure culture proves to limit progress, it will be worth revisiting the question of establishing standards for enrichment cultures.

### “Which genome belongs to which organism? We have this one new trick”

Many talks featured microbial genomes identified in metagenomes and stressed the importance of genome-resolved metagenomics for studying MDM. As composite sequences, though, genomes assembled from shotgun metagenomic sequencing do not capture the biology of a single cell. To address this gap, presenters discussed recent innovations for high-throughput profiling of individual microbes and microbial behaviors. Using a reverse genomics approach, antibodies to predicted cell surface proteins are used to select bacteria of interest from a community for isolation (43). Dr. Tanja Woyke talked about a return to single-cell genomics by using a scalable semi-permeable capsule approach in conjunction with improved whole-genome amplification (30) for higher-quality genomes; not only do single-cell genomes resolve strain-level heterogeneity in populations and provide experimental links to mobile genetic elements, as shown in a hot spring previously (44); they genomically capture taxa missed through metagenomics. Dr. Ben Auch showed how proximity ligation can be used to map mobile genetic element-host interactions across environments and conditions and how Phase Genomics is using their database of such interactions to track antibiotic resistance (45) and engineer endolysin therapies for precision antimicrobials (46).

### “There’s no silver bullet for fine details”

Whether identified in extreme environments or a model organism, microbial genomes contain functional dark matter, referring to the lack of protein annotations for their coding sequences. A number of talks at the meeting focused on addressing this interpretation of MDM. Keynote speaker Dr. Curtis Huttenhower provided a working definition for function in this context, “a biological process specific enough to be confirmed or refuted through laboratory experiments while also general enough to reasonably expect high-throughput assays to provide relevant information.” He highlighted three computational methods developed in his lab: MetaWIBELE (47), FUGAsseM (48), and MACARRoN (49), and showed how integration of multiple data modalities can reveal protein and chemical function where matching to reference databases alone does not. Taking a different approach to function annotation, Dr. Libusha Kelly showed how large language model representations of viral proteins can be leveraged to expand homology detection (50). Both acknowledged that predictions favor microbes that are more frequently encountered in databases and bulk assay experiments, leaving the MDM as the largest reservoir for functional dark matter.

### “Life acts in ways we did not know it could”

Function can also be considered at the organism level. For those who study environmental microbiology, one of the most important functions is respiration. Dr. Emil Ruff described the production of dark oxygen, meaning biotic generation of molecular oxygen without sunlight, by microbes in diverse groundwaters, apparently creating aerobic niches in otherwise anoxic environments (51). Dr. Magdalena Osburn showed that energy metabolism and nutrient cycling are functional differences between core and variable microbes found in distinct geochemical niches sampled over time in the Deep Mine Microbial Observatory. Replication is another function crucial to understanding MDM. Dr. Karen Lloyd described how ancient microbes might remain alive by not expending energy to reproduce themselves (26). Dr. Elizabeth Trembath-Reichert described how temperature, wind, humidity, and other environmental conditions are important to understanding microbes present in the atmosphere, an important emerging ecosystem in its own right. Capturing the *in situ* function of a microbe requires linking its functional potential to the environment where it is active, which requires innovations in geochemical techniques so that more parameters can be measured *in situ*.

Crucial to these functional characterizations was incorporating geochemical information to the experimental design. One of the discussions at the meeting was focused on how to encourage the community to capture geochemical data at the time of microbial sampling. What is the minimal information that should be captured? How should it be reported? Can this be automated somehow? What lessons can be learned from efforts like the National Microbiome Data Collaborative (52), including a mobile application for streamlined metadata collection and Strengthening The Organization and Reporting of Microbiome Studies (53). As environmental sampling continues to grow, it is imperative that we address this important gap in our community.

### “We should talk”

The symposium featured a mix of wet and dry lab talks. More than once an experimentalist brought up a challenge they are facing with their data analysis and a bioinformatician in the room suggested they discuss further. Dr. Greg Caporaso spoke about a decade of developing, teaching, supporting, and applying QIIME 2 (54). He highlighted the community forum, multi-level documentation, and automated data provenance recording as enduring strengths of the platform and models for the development of next-generation bioinformatics software that will support ethical and trustworthy inference from machine learning and artificial intelligence technologies in microbiome science. The meeting provided a space for data scientists and experimentalists to connect so that each could update the other on new methods, findings, and open questions. This exchange underscored how progress in microbial research increasingly depends on a dialog between generating biological observations and developing the computational tools to help translate them into insights.

### “Trust biology”

By the end of 2 days, it was clear that nature holds many surprises, especially in the context of MDM. Of course we must question what we see in our experiments, be concerned about contamination, and be diligent in our utilization of databases and analysis software as was repeated throughout the talks. Yet time and again, scientists showed that their findings challenged previous assumptions and reshaped our current understanding. When asked about his first encounter with MDM, Dr. Simon Roux told a story about a viral genome he analyzed in graduate school that appeared to contain genes from both DNA and RNA viruses. Assuming this to be an assembly error, he dismissed it. A little later, another group led by Prof. Ken Stedman showed with PCR that it was in fact a single virus, providing evidence of lateral gene exchange between DNA and RNA viruses (55). Environmental microbes are a wellspring for novel biology; we just need to keep our eyes and minds open to see it.

## EMERGING FRONTIERS AND OPPORTUNITIES

### Ecological discoveries and environmental function

- Oxygen production in permanently dark environments creates unexpected aerobic niches.
- Atmospheric microbiomes are structured, diverse, and ecologically relevant.
- Cryptic element cycles are widespread, but their ecological and biogeochemical impact remains largely unknown.
- Subsurface aquifers and the human gut have little in common, yet are governed by similar ecological mechanisms.
- (Geo)chemical context is critical to understand how microbes contribute to any ecosystem.

### Methods and characterization

- Culture-based techniques and reverse genomics approaches can be used to characterize members of the microbial dark matter.
- Capsule-based single-cell methods reveal overlooked microbial players.
- Functional annotation and characterization of proteins encoded in metagenomes remains a key frontier for both computational and AI-driven approaches.

## FUTURE DIRECTIONS

- Refine cultivation standards to encourage organized sharing of mixed cultures. Although such cultures were previously considered impure or dirty, they actually enable cultivation of MDM members that cannot be isolated.
- Develop highly efficient nucleic acid extraction techniques and contaminant-free reagents to achieve complete metagenome recovery without introducing unwanted DNA or RNA.
- Enable low-barrier collection and archival of geochemical data from microbial sampling.
- Establish universal frameworks for functional annotation.
- Expand single-cell genomics and proximity-ligation tools into clinical microbiome research.
- Build microbial field guides with predictive ecological and functional attributes.
- Encourage open data and community science models to accelerate discovery.
- Develop planetary protection protocols informed by evidence of microbial persistence in space.

## CONCLUSION

The Microbial Dark Matter Symposium exemplified the spirit of interdisciplinary inquiry. Researchers across fields are united by a common goal of illuminating the microbial life that has evaded detection and understanding for decades. Whether lurking in the sequence data of samples from our everyday lives, embedded deep in Earth’s crust, drifting in the atmosphere, or clinging to spacecraft, MDM continues to challenge the limits of our scientific imagination.
